# Mild Hypothermia May Offer Some Improvement to Patients with MODS after CPB Surgery

**DOI:** 10.5935/1678-9741.20160048

**Published:** 2016

**Authors:** Xiaoqi Zhao, Tianxiang Gu, Zongyi Xiu, Enyi Shi, Lei Yu

**Affiliations:** 1 Department of Cardiac Surgery ICU, The First Affiliated Hospital of China Medical University, Shenyang, P.R. China

**Keywords:** Hypothermia, Multiple Organ Failure, Shock, Cardiogenic

## Abstract

**OBJECTIVE:**

To summarize the effect of mild hypothermia on function of the organs in patients with multiple organ dysfunction syndrome after cardiopulmonary bypass surgery.

**METHODS:**

The patients were randomly divided into two groups, northermia group (n=71) and hypothermia group (n=89). We immediately began cooling the hypothermia group when test results showed multiple organ dysfunction syndrome, meanwhile all patients of two groups were drawn blood to test blood gas, liver and kidney function, blood coagulation function, and evaluated the cardiac function using echocardiography from 12 to 36 hours. We compared the difference of intra-aortic balloon pump, extracorporeal membrane oxygenation rate and mortality within one month after intensive care unit admission.

**RESULTS:**

Among the 160 patients, 36 died, 10 (11.24%) patients were from the hypothermia group and 26 (36.6%) from the northermia group (*P* <0.05). In northermia group, 45 (63.38%) patients used intra-aortic balloon pump and 4 (5.63%), extracorporeal membrane oxygenation; in hypothermia group, 35 (39.32%) patients used intra-aortic balloon pump and 2 (2.25%), extracorporeal membrane oxygenation（ *P* <0.05). The patients' heart rate decreased significantly in the hypothermia group. The heart rate of hypothermia group is significantly slower than the northermia group at the 36^th^ hour (*P* <0.05). But the mean arterial pressure of hypothermia group is significantly higher than the northermia group at the 36^th^ hour (*P* <0.05). In hypothermia group, PO_2_, SvO_2_ and lactate were improved significantly compared to pre-cooling (*P* <0.05), and they were significantly better than the northermia group at the 36^th^ hour (*P* <0.05%). Prothrombin time and activated partial thromboplastin time have no significantly difference between the two groups (*P* >0.05). But the platelet count has significantly difference between the two groups at the 36^th^ hour (*P* <0.05). The aspartate transaminase, alanine transaminase and creatinine were improved significantly in the hypothermia group, and they were significantly better than the northermia group (*P* <0.05).

**CONCLUSION:**

Mild hypothermia is feasible and safe for patients with multiple organ dysfunction syndrome after cardiopulmonary bypass surgery.

**Table:** 

**Abbreviations, acronyms & symbols**	
ALB	= Albumin
ALT	= Alanine transaminase
APTT	= Activated partial thromboplastin time
AST	= Aspartate transaminase
ATP	= Adenosine triphosphate
BSA	= Body surface area
CI	= Cardiac index
CPB	= Cardiopulmonary bypass
ECMO	= Extracorporeal membrane oxygenation
Emax	= Ventricular end-systolic maximum elastance
HR	= Heart rate
HT	= Hypothermia group
IABP	= Intra-aortic balloon pump
ICU	= Intensive Care Unit
LCOS	= Low cardiac output syndrome
MAP	= Mean arterial pressure
MODS	= Multiple organ dysfunction syndrome
NT	= Northermia group
PLT	= Platelet count
PT	= Prothrombin time
PVA	= Pressure-volume area
SIRS	= Systemic inflammatory response syndrome
SV	= Stroke volume

## INTRODUCTION

Multiple organ dysfunction syndrome (MODS) is defined as "the presence of altered organ function in an acutely ill patient such that homeostasis cannot be maintained with intervention"^[1]^. It is a cause of high mortality and morbidity in Intensive Care Unit (ICU)^[2]^. Many advances have been made in the treatment of MODS. Hypothermia has become an established therapeutic concept in the treatment of cardiovascular and neurological diseases^[3]^, and it has shown myocardial and neurological protection, yet the benefits for MODS after cardiac surgery have not been well defined. We hypothesized that mild hypothermia has organ protective effects and can ameliorate organ dysfunction to improve the survival rate for the patients with MODS after cardiopulmonary bypass (CPB) cardiac surgery.

## METHODS

### Study Design

The study was designed as prospective unblended intervention trial where patients served as their own controls. This study protocol was approved by the ethics committee of the First Affiliated Hospital of China Medical University. The patients with MODS after cardiac surgery underdoing CPB from May 2011 to February 2015 were screened for this study. The cardiac function of all the patients was class II-III (New York Heart Association).

Exclusion criteria: patients with respiratory, nervous, hematological system diseases or liver, kidney, digestive system diseases before surgery; patients with massive blood transfusion (massive blood transfusion defined as the replacement of a patients' total blood volume in less than 24 hours, or as the acute administration more than half the patients' estimated blood volume per hour); patients died within 24 hours after ICU admission.

The MODS diagnosis criterion of MODS^[4]^ is shown in Table 1.

**Table 1:** The diagnostic criteria of MODS^[4]^.

**System**	**Criterion**	
Circulatory	(1) SBP <90 mmHg; (2) MAP <70 mmHg; (3) shock, ventricular tachycardia or ventricular fibrillation, myocardial infarction	Any of the three
Respiratory	Oxygenation index (PO/FiO_2_) <300 mmHg	
Nervous	(1) Indifference or agitation, drowsiness, coma (2) Glasgow score ≤ 14	Any of the two
Blood	(1) PLT<100*10^9^/L (2) TT, APTT Prolonged or shortened PT,3P(+)	Any of the two
Liver	(1) TBIL >20.5 µmol/L (2) ALB <28 g/L;	Any of the two
Urinary system	(1) blood Cr>123.8 µmol/L; (2) urine volume < 500 ml/24h.	Any of the two
Gastrointestinal	(1) bowel sounds weaken or disappear; (2) gastric drainage fluid or stool occult blood (+), or black stool, haematemesis. (3) intra-abdominal pressure ≥ 11 cmH_2_O.	Any of the three

ALB=albumin; APTT=activated partial thromboplastin time; Cr=creatinine; MAP=mean arterial pressure; PLT=platelet count; PT=prothrombin time; SBP=systolic blood pressure; TBIL=total bilirubin; TT=thrombin time

All the patients used urine catheter with temperature probe by which we can monitore the patients' bladder temperature. At the admission to Cardiac Surgery ICU, the patients were assisted by ventilator, tidal volume (ml) = Body weight (kg)*10(ml/kg); respiratory frequency 12-14 times/min; FiO_2_:0.50-0.75. At the same time, intravenous sedation and muscular relaxation agent were infused. We used the echocardiography to monitor cardiac stroke volume (SV), then we calculated cardiac index (CI), CI=heart rate (HR)*SV/body surface area (BSA).

The basic treatments for the patients of the two groups are same, including intravenous infusion of vasoactive agents to maintain hemodynamic stability and improve organs function. However, in the hypothermia group (HT), hypothermia treatment was implemented by a computer cooling blanket (CJ1 temperature lowering instrument). The patient was placed on the blanket which is filled with the variable temperature cycle of cryogenic mat. We adjusted the temperature of cooling blanket until the patients' bladder temperature reached and maintained 35ºC. The cooling rate was about 1.0ºC/h^[5]^. The bladder temperature was reduced from 36.1ºC±0.2ºC to 33.1ºC±0.1ºC within 168±10min of starting cooling and remained lowered at 32.9±0.5ºC during 36 hours.

### Data Collection

If patients' postoperative test results (we marked it as precooling) showed two or more abnormal organ function, we considered the existence of MODS. The patients were randomly divided into two groups, one is northermia group (NT) n=71, the other one is HT (hypothermia group) n=89. Then we immediately began cooling the HT (we marked the time as 0h), meanwhile all patients were drawn arterial blood and venous blood to test blood gas, liver and kidney function, blood coagulation function, and evaluated the cardiac function using echocardiography from 12 to 36 hours. We monitored each patient closely about the parameters of vital signs: mean arterial pressure (MAP), HR, urinary volume.

### Statistical Analysis

All statistical analysis were performed using SPSS 17.0, measurement data of each group were performed using normality test, F-test and tested with Rank test.

## RESULTS

**Fig. 1:**
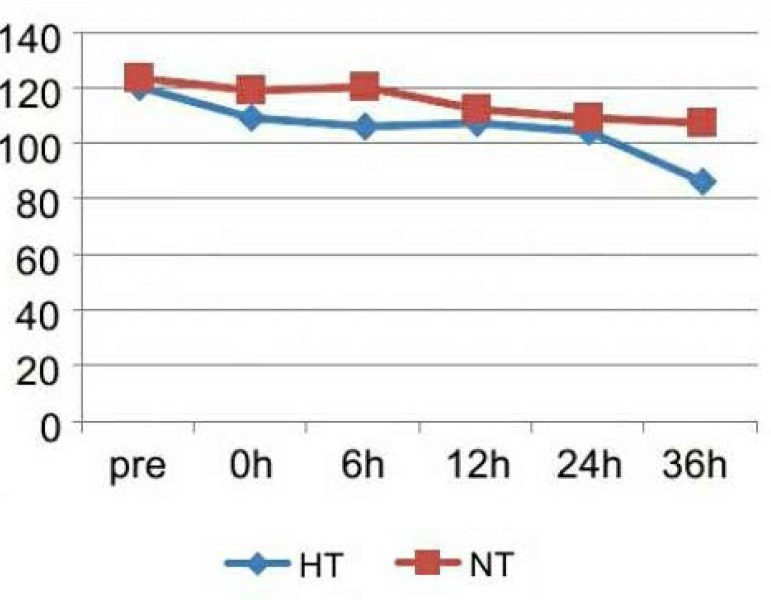
Heart rate (HR) changes of the two groups. The patients’ heart rate decreased significantly after the application of hypothermia. The HR difference between two groups at the 36^th^ hour is significant (P<0.05).

**Fig. 2:**
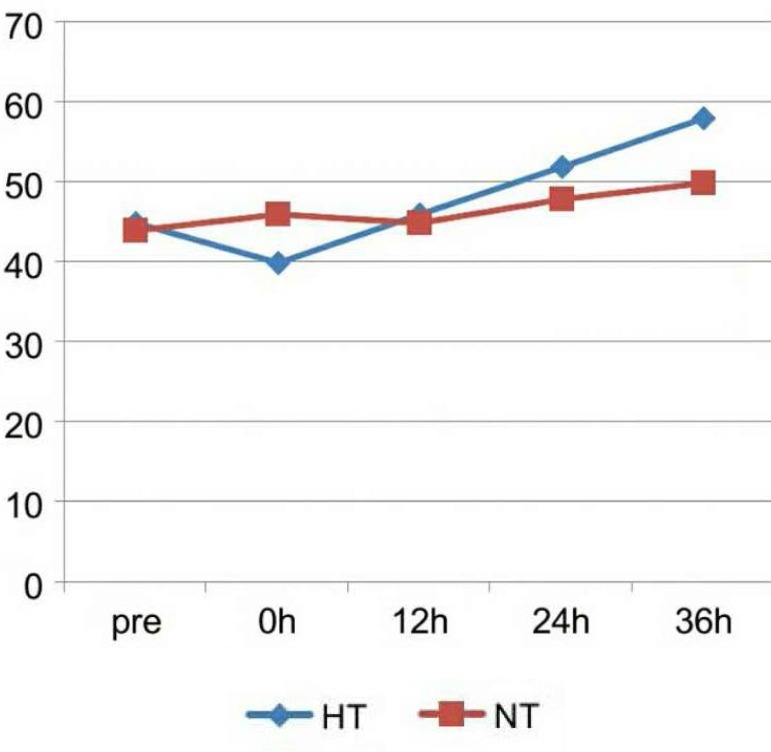
Mean arterial pressure (MAP) changes of the two groups. The MAP of the HT is significantly lower than the NT at 0 hour, because we used the sedation and neuromuscular blockade. But the MAP of the HT is significantly higher than the NT after hypothermia at the 36^th^ hour (P<0.05).

Among the 160 patients considered for inclusion, 36 died, 10 (11.24%) patients were from the HT and 26 (36.6%) were from the NT (*P* <0.05). In NT, there were 45 patients who used IABP = Intra-aortic balloon pump (63.38%), 4 patients who used extracorporeal membrane oxygenation (ECMO) (5.63%). In HT, there were 35 patients who used IABP (39.32%), 2 (2.25%) patients ECMO (*P* <0.05). The patients' HR decreased significantly after the application of hypothermia. The HR difference between the two groups at the 36^th^ hour is significant (*P* <0.05), shown as Figure 1. The MAP of HT is lower than NT significantly at 0 hour, because we used the sedation and muscular relaxation agent, and the depth of sedation in HT is much more deeply than NT in order to prevent chill. But the MAP of HT is significantly higher than NT after hypothermia at the 36^th^ hour (*P* <0.05), shown as Figure 2. PO_2_, SvO_2_, lactate and CI have no significantly difference between the two groups pre-cooling (*P* >0.05). In HT group, PO_2_, SvO_2_ and lactate were improved significantly at the 36^th^ hour compared with pre-cooling (*P* <0.05), and they were better than NT group significantly at the 36^th^ hour (*P* <0.05%) as shown in Table 2. Prothrombin time (PT) and activated partial thromboplastin time (APTT) have no significantly difference between the two groups (*P* >0.05). But the platelet count (PLT) has significantly difference between the two groups at the 36^th^ hour (*P* <0.05%), shown in Table 3. The aspartate transaminase (AST), alanine transaminase (ALT) and creatinine were improved significantly in the HT group, and they were significantly better than the NT group (*P* <0.05), shown in Table 4.

**Table 2:** Changes of blood gas and CI.

**Variables**	**HT (n=89)**				**NT (n=71)**			
	pre-cooling	12h	24h	36h	pre-cooling	12h	24h	36h
PO_2_ (mmHg)	88±13	89±17	95±9	102±11#*	85±15	88±10	90±12	89±14
SvO_2_ (%)	41±3.0	44±2.5	50±1.4	53±1.1#*	43±2.9	42±2.0	45±2.3	48±1.7
Lac (mmol/L)	8.2±2.6	8.9±2.0	8.0±1.1	5.5±1.5#*	7.9±2.7	8.5±3.0	8.2±1.9	8.1±1.8
CI (ml.m^-1^.m^-2^)	30±5.0	34±4.6	37±5.2	42±5.0#*	30.2±4.5	32±2.9	31±4.1	34±2.8

# *P*<0.05 *versus* pre-cooling;

* *P*<0.05 *versus* NT

CI=cardiac index; Lac=lactate

**Table 3:** Coagulation index changes.

**Variables**	**HT (n=89)**				**NT (n=71)**			
	pre-cooling	12h	24h	36h	pre-cooling	12h	24h	36h
PT(s)	16.0±3.0	15.9+3.9	15.0+2.6	14.4+3.5	14.5+3.1	13.6+1.6	13.4+2.1	13.6+1.9
APTT(s)	38.9±6.8	41.1+6.8	36.3+4.0	40.0+11.2	38.1+5.7	38.8+4.5	36.7+4.5	38.3+4.1
PLT	259+101	218+158	267+168	372+150#*	261+99	247+185	234+178	300+139

# P<0.05 *versus* pre-cooling;

* P<0.05 *versus* NT.

APTT=activated partial thromboplastin time; PLT=platelet count; PT=prothrombin time

**Table 4:** Liver and kidney index changes.

**Variables**	**HT (n=89)**		**NT (n=71)**	
	pre-cooling	36h	pre-cooling	36h
rank (ALT)	341	257	321	398
rank (AST)	338	268.1	318	370.8
rank (Cr)	388	246.5	364	379.5

ALT=alanine transaminase; AST=aspartate transaminase; Cr=creatinine

## DISCUSSION

The first major finding of the present study is that the induction of mild hypothermia until the 36^th^ hour is feasible and safe for patients with MODS after CPB. There are several proposed mechanisms for the development of MODS, including^[6]^:(1) cell or tissue hypoxia; (2) induction of cellular apoptosis; (3) translocation of microbes or components of microbes from the gastrointestinal tract; (4) immune system dysregulation, and (5) mitochondrial dysfunction. The predominant failure organs involved in MODS are hepatic, respiratory, gastrointestinal, cardiovascular, coagulation, renal, central nervous and endocrine systems^[7]^. Most cardiac surgery need CPB, but intra-operation of cardiac surgery, CPB can lead to a variety of inflammatory medium content increased significantly. As a result abnormal cytokine expression and systemic inflammatory reaction in tissues impaired the organs function^[8]^, and even lead to further multiple organ dysfunction^[9]^.

Hypothermia can be divided into mild hypothermia (32-35ºC), moderate hypothermia (28-32ºC), deep hypothermia (20-28ºC), super-deep hypothermia (<20ºC)^[10]^. Many studies have shown that mild hypothermia interference with the body's homeostasis is not significant. It can decrease the oxygen consumption of the tissue, delay the adenosine triphosphate (ATP) consumption when tissue is ischemia^[11]^. The mild hypothermia can improve the organization of ischemia hypoxia tolerance^[12]^, so that the organization oxygen can achieve balance between supply and demand, maintain each organ function. It reduces metabolic demand and high energy phosphate utilization in the myocardium^[13-15]^, it is advantageous to oxygen uptake and utilization of the heart cells. At the same time, mild hypothermia can avoid ventricular fibrillation which may be caused by deep hypothermia, and Bernard et al.^[16]^ have discovered the incidence of infection is not common in short term hypothermia treatment (12-36 hours), so we chose the mild hypothermia for 36 hours in this study.

Some studies have shown that mild hypothermia inhibits inflammation reaction, inhibit the release and expression of TNF-alpha and ICAM-1, and protect the organ function. In the hypothermia application process, it should be fully realized the side effect as its influence on blood coagulation function and so on. But in our study, we did not observe any complication of clinical relevance associated with mild hypothermia, especially bleeding, thrombosis, arterial or pulmonary embolism and no adverse haemodynamic events. Rodriguez et al.^[17]^ showed that, in the case of patients with chill, oxygen consumption will increase by 45%. Frank et al.^[18]^ found that for patients with a history of myocardial ischemia, chill increased the risk of myocardial infarction, so we should use sedation and neuromuscular blockade to anti-shiver in the duration of hypothermia.

The second major finding is that moderate hypothermia significantly improves parameters of organ function in the patients with MODS after CPB surgery. Low cardiac output syndrome (LCOS) is a predominant cause of the MODS after cardiac surgery^[19]^, meanwhile, MODS may lead to cardiovascular dysfunction characterized by biventricular dilatation, decreased ejection fraction and hypotension^[20]^. Then MODS may lead to form a vicious circle, and make the condition worse. So, in order to treat the MODS after cardiac surgery, correcting heart function effectively is the most important. Because of the improvement of cardiac function, the perfusion of the other organs was improved and the organ functions were improved further. As cardiac power output predicts mortality during LCOS^[21]^, our data indicate that the improvement to the patients with MODS after CPB surgery in the hypothermia group may in part be related to improved cardiac performance. In a situation of severely depressed left ventricle function, cooling may improve systemic oxygen supply-demand balance not only by reducing demand but also by increasing cardiac output via its positive inotropic effect^[22]^. The positive inotropic effect of hypothermia has been confirmed by Gotberg et al.^[23]^ using whole-animal models of cardiogenic shock. The inotropic effect of hypothermia is associated with an effect at the level of myofilaments^[24]^, without causing changes in sarcoplasmatic calcium content or intracellular calcium concentration^[25]^. This implies that hypothermia can recruit a contractile reserve without increasing energy demand. In the acute myocardial infarction model, therapeutic hypothermia has been proved that it improves myocardial dysfunction by reducing ischemia-reperfusion injury and results in a decreased size of infarction^[26,27]^. Ristagno et al.^[28]^ studied the effect of hypothermia on ventricular myocyte contractility, and discovered that hypothermia increased ventricular myocyte contractility either under conditions of normal perfusion or after perfusion following a 10 min interval of ischemia. Shattock & Bers^[29]^ and Miao & Lynch^[30]^ showed that hypothermia increases in myocardial force generation and this was confirmed by Fukunami & Hearse^[31]^ and Nishimura et al.^[32]^. Suga et al.^[33]^ resported that cardiac cooling increased ventricular end-systolic maximum elastance (Emax) without affecting systolic pressure-volume area (PVA)-independent VO_2_ and has energetically more advantages in saving myocardial oxygenconsumption compared to catecholamine in cross circulated canine heart preparations.

Some limitations of our study should be acknowledged. Firstly, this study was designed as a prospective unblended intervention trial, so there is a chance that sicker patients might have been considered for normothermia. But in our study, the initial parameters have no significant differences between the two groups. So the authors think the potential bias induced by that is limited. Another limitation is the information on temperature. We recorded the time in which TH was reached, but we didn't document the course of the temperature during rewarming. Thus, maybe further research is needed to identify the effect of the course of rewarming on mortality.

## CONCLUSION

In summary, our studies demonstrate that mild hypothermia is feasible and safe also for patients with MODS after CPB surgery. Mild hypothermia can improve the organ function effectively, and improve the morbidity and mortality of the patients. It can slow the MODS/Systemic inflammatory response syndrome (SIRS) development speed and reduce the time of protection and further treatment for cells and organs.

**Table:** 

**Authors' roles & responsibilities**	
XZ	Final manuscript approval
TG	Conception and design study; final manuscript approval
ZX	Statistical analysis; final manuscript approval
ES	Manuscript redaction or critical review of its content; final manuscript approval
LY	Statistical analysis; final manuscript approval
